# Supplementary material for: A genome assembly and the somatic genetic and epigenetic mutation rate in a wild long-lived perennial Populus trichocarpa
Source: Genome Biol. 2020 Oct 6;21:259. doi: 10.1186/s13059-020-02162-5 (PMC7539514; doi:10.1186/s13059-020-02162-5)
Supplement: Supplementary file 3 — Additional file 3. Supplementary text. Includes an expanded description of how epimutation rates were estimated. [file 13059_2020_2162_MOESM3_ESM.pdf]

mutSOMA: Estimating somatic mutation rates from high-throughput sequencing data in trees

We developed *mutSOMA*, a computational method for estimating somatic mutation rates from high-throughput sequencing data in trees. The method treats the tree branching structure as an intra-organism phylogeny of somatic lineages. Its analytical framework builds on ideas introduced in van der Graaf et al. (2015) and Shahryary et al. 2019 (co-submission). Software implementing the method can be found at (https://github.com/jlab-code/mutSOMA).

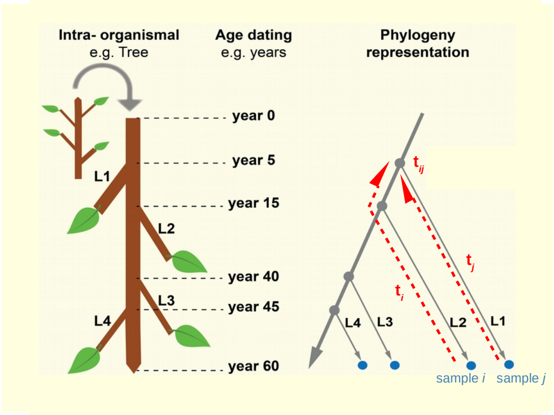

Figure 1: Long-lived perennials, such as trees, can be viewed as a natural mutation accumulation system. In this case, the tree branching structure can be treated as an intra-organismal phylogeny of somatic lineages that carry information about the mutational history of each branch. Re-sequencing data is obtained from leaf samples of selected branches. *mutSOMA* uses the genotype data of the samples along with the coring data to estimate the per year rate of somatic mutations. L1, L2, L3, L4 denote the branches of the tree; blue circles denote sequenced samples; grey circles denote branch points. Highlighted are samples *i* and *j* and the corresponding branch ages t_i, t_j as well as the age of the most recent common branch point t_{ij}.

Calculating genetic divergence

We start from the variant calls (i.e. .vcf files) obtained from different branches of the tree (**Figure 1**). For the i-*th* sample ($i = 1, \dots, M$) we let g_{ik} be the observed genotype at the k-*th* locus ($k = 1, \dots, N$), where N is the effective genome size (i.e. the total number of bases with sufficient coverage). With four possible nucleotides (A, C, T, G), g_{ik} can have 16 possible genotypes in a diploid genome, 4 homozygous (A|A, T|T, C|C, G|G) and 12 heterozygous (A|G, A|T, $\dots$, G|C). The $\cdot|\cdot$ notation refers to the

nucleotide on the forward (+) strand on each of the two homologous chromosomes. Using this coding, we calculate the genetic divergence, D, between any two samples i and j as follows:

$$D_{ij} = \sum_{k=1}^{N} I(g_{ik}, g_{jk}) N^{-1}, \tag{1}$$

where $I(\cdot)$ is an indicator function, such that, $I(\cdot) = 0$ if the two samples share no alleles at locus k (e.g. A|A and G|G), 0.5 if they share one (e.g. A|A and A|G), and 1 if they share both alleles (e.g. A|A and A|A). We suppose that D_{ij} is related to the developmental divergence time of samples i and j through a somatic mutation model. The divergence times (in years) are calculated from the coring data (**Figure 1**).

Modelling age-dependent genetic divergence

We model the time-dependent genetic divergence between samples using

$$D_{ij} = c + D^{\bullet}_{ij}(M_{\Theta}) + \epsilon_{ij}. \tag{2}$$

Here $\epsilon_{ij} \sim N(0, \sigma^2)$ is the normally distributed residual error, c is the intercept, and $D^{\bullet}_{ij}(M_{\Theta})$ is the expected genetic divergence between samples i and j as a function of an underlying mutation model $M(\cdot)$ with parameter vector Θ. Parameter vector Θ contains the unknown mutation rate γ and the unknown level of heterozygosity δ of the 'founder cells' of the tree (**see Figure 1**). The estimation of the residual variance in the model accounts for the fact that part of the observed genetic divergence between any two samples is driven by genotyping errors. We have that

$$\begin{aligned} D^{\bullet}_{ij}(M_{\Theta}) &= \sum_{n \in v} \sum_{l \in v} \sum_{m \in v} I(l, m) \\ &\cdot \; Pr(g_{ik} = l, g_{jk} = m | g_{ijk} = n, M_{\Theta}) \\ &\cdot \; Pr(g_{ijk} = n | M_{\Theta}), \end{aligned}$$

where g_{ijk} is the genotype at the k locus of the the most recent progenitor cells that are developmentally shared between samples i and j, and $v \in \{\text{A|A, T|T, C|C, } \ldots \text{, G|T}\}$. Since the two samples are conditionally independent, we can further write:

$$Pr(g_{ik}, g_{jk}|g_{ijk}, M_\Theta) = Pr(g_{ik}|g_{ijk}, M_\Theta) \cdot Pr(g_{jk}|g_{ijk}, M_\Theta).$$

To be able to evaluate these conditional probabilities it is necessary to posit an explicit form for the somatic mutation model, M_Θ. To motivate this, we define $\mathbf{G}_{(16\times16)}$ to be a 16×16 transition matrix, which summarizes the probability of transitioning from genotype l to m in the time interval $[t, t+1]$. $\mathbf{G}$ can be written in the following partitioned form:

$$\mathbf{G}_{(16\times16)} = \left(\begin{array}{c|c} \mathbf{T1}_{(4\times4)} & \mathbf{T2}_{(4\times12)} \\ \hline \mathbf{T3}_{(12\times4)} & \mathbf{T4}_{(12\times12)} \end{array}\right)$$

where sub-matrices **T1**, **T2**, **T3** and **T4** contain the transition probabilities between homozygous to homozygous, homozygous to heterozygous, heterozygous to homozygous and heterozygous to heterozygous genotypes, respectively. Explicit elements of each of these matrices can be worked out and hold for both somatic and clonally propagated systems. As there is no genetic segregation, the elements of this matrix are only governed by the mutation rate γ. For instance, symmetrical sub-matrix **T1** is

$$\begin{array}{cc} & \begin{array}{cccc} \text{A|A (t+1)} & \text{C|C (t+1)} & \text{T|T (t+1)} & \text{G|G (t+1)} \end{array} \\ \mathbf{T1}_{(4\times4)} = & \begin{bmatrix} (1-\gamma)^2 & \frac{1}{9}\gamma^2 & \frac{1}{9}\gamma^2 & \frac{1}{9}\gamma^2 \\ \cdot & (1-\gamma)^2 & \frac{1}{9}\gamma^2 & \frac{1}{9}\gamma^2 \\ \cdot & \cdot & (1-\gamma)^2 & \frac{1}{9}\gamma^2 \\ \cdot & \cdot & \cdot & (1-\gamma)^2 \end{bmatrix} \begin{array}{c} \text{A|A (t)} \\ \text{C|C (t)} \\ \text{T|T (t)} \\ \text{G|G (t)} \end{array} \end{array}$$

, and **T4** is

$$\mathbf{T4}_{(12\times 12)} = \begin{array}{c} \begin{array}{cccc} \text{A|C (t+1)} & \text{A|T (t+1)} & \cdot & \text{G|T (t+1)} \end{array} \\ \left[\begin{array}{cccc} (1-\gamma)^2 & \frac{1}{3}(1-\gamma)\gamma & \cdot & \frac{1}{9}\gamma^2 \\ \cdot & \cdot & \cdot & \cdot \\ \cdot & \cdot & \cdot & \frac{1}{9}\gamma^2 \\ \cdot & \cdot & \cdot & (1-\gamma)^2 \end{array}\right] \begin{array}{l} \text{A|C (t)} \\ \text{A|T (t)} \\ \cdot \\ \text{G|T (t)} \end{array} \end{array}$$

.

Based on Markov chain theory, the conditional probability $Pr(g_{ik}|g_{ijk}, M_\Theta)$ can then be expressed in terms of **G** as follows:

$$\sum_{n\in v} Pr(g_{ik} = v_r|g_{ijk} = n, M_\Theta) = \sum_{s=1}^{16} \left[\mathbf{G}^{t_i - t_{ij}}\right]_{rs}$$

where $r = 1, \ldots, 16$ is a fixed index corresponding to genotype vector {A|A, C|C, …, G|T }, t_i is the age of sample i and t_{ij} is the age of the most recent common branch point of samples i and j, $(t_{ij} \leq t_i, t_j)$. Expressions for $Pr(g_{jk}|g_{ijk}, M_\Theta, t_j)$ can be derived accordingly, by simply replacing t_i by t_j in the above equation. Note that the calculation of these conditional probabilities requires repeated matrix multiplication. However, a direct evaluation of these equations is also possible using the fact that

$$\mathbf{G}^{t_i - t_{ij}} = \mathbf{p}\mathbf{V}^{t_i - t_{ij}}\mathbf{p}^{-1} \text{ and } \mathbf{G}^{t_j - t_{ij}} = \mathbf{p}\mathbf{V}^{t_j - t_{ij}}\mathbf{p}^{-1},$$

where **p** is the eigenvector of matrix **G** and **V** is a diagonal matrix of eigenvalues. Finally, to derive $D^\bullet_{ij}(M_\Theta)$, we also need to supply $Pr(g_{ijk} = n|M_\Theta)$; that is, the probability that any given locus k in most recent shared progenitor cells of i and j is in state n ($n \in$ {A|A, C|C, …, G|T }). To do this, consider the genome of the hypothetical founder cell of tree at time $t = 1$, and let $\pi = [p_1\ p_2\ p_3\ \ldots\ p_{16}]$ be a row vector of probabilities corresponding to 16 possible genotypes, respectively. Using

Markov Chain theory we have

$$Pr(g_{ijk} = v_r | M_\Theta) \quad = \quad \left[\pi\, \mathbf{G}^{(t_{ij}-1)}\right]_r .$$

Assuming that *Populus trichocarpa* genome is at an evolutionary mutation equilibrium, we can obtained the probability elements of vector π as follows

$$p_1 = \frac{x(A|A)}{N}(1-\delta), \qquad p_2 = \frac{x(C|C)}{N}(1-\delta), \qquad \ldots, \qquad p_{16} = \frac{x(G|T)}{12N}\delta$$

where $\delta \in [0,1]$ is the overall level of heterozygosity in the genome, $x(\cdot)$ is the frequency count of the loci with that particular genotype, and N is the effective genome size.

Model inference

To obtain estimates for Θ, we seek to minimize

$$\nabla \textstyle\sum_{q=1}^{M} \left(D_q - D_q^{\bullet}(M_\Theta) - c\right)^2 = \mathbf{0}, \tag{3}$$

where the summation is over all M unique pairs of sequenced samples in the pedigree. Minimization is performed using the "Nelder-Mead" algorithm as part of the optimx package in R.

Confidence intervals

We obtain confidence intervals for the estimated model parameters by boostrapping the model residuals. The procedure has the following steps: 1. For the qth sample pair q ($q = 1, \cdots, M$) we define a new response variable $B_q = \hat{D}_q + \hat{\epsilon}_k$, where $\hat{D}_q$ is the fitted divergence for the qth pair, and $\hat{\epsilon}_k$ is drawn at random and with replacement from the $1 \times M$ vector of fitted model residuals; 2. Refit the model using the new response variable, and obtain estimates for the model parameters. 3. Repeat steps 1. to 2. a large number of times to obtain a boostrap distribution. 4. Use the bootrap

distribution from 3. to obtain empirical confidence intervals.
